# Microstructure of Milled Polyacrylonitrile-Based Carbon Fiber Analyzed by Micro-Raman Spectroscopy and TEM

**DOI:** 10.3390/ma14164711

**Published:** 2021-08-20

**Authors:** Sang-Hye Lee, Sang-Min Lee, Jae-Seung Roh

**Affiliations:** 1 School of Materials Science and Engineering, Kumoh National Institute of Technology, 61 Daehak-ro, Gumi 39177, Korea; shlee3106@kumoh.ac.kr; 2 Carbolab Co., Ltd., Gumi 39425, Korea; dltkdals3232@hanmail.net

**Keywords:** crystallinity, milled carbon fiber, PAN-based carbon fiber, Raman, XRD

## Abstract

Milled polyacrylonitrile (PAN)-based Carbon Fibers (mPCFs) were prepared from PAN-based carbon fibers by using a ball milling process. The resulting structural changes in the mPCFs were analyzed by correlating the analytical results obtained by X-ray diffraction (XRD) and Raman spectroscopy and verified by transmission electron microscopy (TEM) lattice images and diffraction patterns. The crystallite size *L_a_* calculated from the XRD measurements decreased as the milling time was increased to 12 h and then decreased as the milling time was further increased to 18 h. The La of both partially-milled Carbon Fiber (pmCF) and milled Carbon Fiber (mCF) calculated from the Raman spectroscopy data continuously increased as the milling time increased. The difference may be because XRD measured the entire sample regardless of pmCF and mCF, while Raman spectroscopy was limited to measuring the surface and differentiated pmCF and mCF. As the ball milling time increased, the fiber surface was firstly broken by the impact energy of the balls, decreasing crystallinity, while the La inside the unbroken fibers increased.

## 1. Introduction

Milled Carbon Fiber (mCF) is a type of carbon fiber with a filament shorter than chopped carbon fiber, typically a length of 1 mm or less [1]. The mCF is generally made from polyacrylonitrile (PAN) and mesophase pitch carbon fibers using various pulverizing methods (ball mill, melt blown, mechanical grinding, etc.). Milled mesophase pitch-based carbon fiber has been studied for application as an anode material in Li-ion batteries [2], and milled PAN-based Carbon Fiber (mPCFs) is mainly used as a filler for polymer composite materials for increasing their conductivity [3,4,5,6,7]. Most studies on mPCFs have focused on their electric conductivity, which depends on the filament length and amount when used as a filler for polymer composite materials [3,4,5,6,7,8]. However, little has been reported about their crystallinity, which is the most important factor affecting electric conductivity.

M. Endo [2] prepared milled mesophase pitch-based carbon fiber by melt-blow and analyzed its crystallinity using XRD and Raman spectroscopy. The XRD results showed that the *d*_002_ and *L_c_* increased as the thermal treatment temperature increased. The Raman analysis showed that *R* (=*I*_1360_*/I*_1580_) decreased and the crystallite *L_a_* increased as the thermal treatment temperature increased. Based on these results, M. Endo reported that the crystallinity of milled mesophase pitch-based carbon fiber could be effectively analyzed by XRD and Raman spectroscopy.

Tuinstra and Koening [9] firstly reported the correlation between *R* and crystallite size *L_a_* using the peak intensity at 1360 and 1580 cm^−1^ by using Raman scattering-based methods for characterizing graphite. After their study, Raman spectroscopy has become one of the most important analytical methods for studying the structural characteristics of carbon and graphite [10,11,12].

In our previous studies, we prepared mPCFs by varying the ball milling time and then analyzed the crystallinity and electrical conductivity with XRD [13]. The asymmetric (002) peak was deconvoluted into two peaks: one for less-developed crystalline carbon (LDCC) and the other more-developed crystalline carbon (MDCC). After milling, the Lc of both LDCC and MDCC was lower, the *d*_002_ was higher, and the LDCC fraction had also increased. In other words, the XRD results showed that mPCFs became more amorphous as the milling time was increased.

We conducted the present study in order to investigate the crystallinity of mPCFs according to milling time by using Raman spectroscopy and analyzed the results in association with the XRD results. Changes in the crystallinity of the mPCFs were also verified by transmission electron microscopy (TEM).

## 2. Experimental

### 2.1. Sample Preparation

The starting carbon fiber used in the present study was PAN-based carbon fiber T700 (12 K) manufactured by Toray, Tokyo, Japan. The starting carbon fiber was inserted to a tube furnace in nitrogen atmosphere and kept at 500 ℃ for 1 h to remove the sizing agent. In the present study, all of the ball milling processes were performed using the desized carbon fiber.

### 2.2. Ball Milling and Shape Observation

The desized carbon fiber was cut to 20 mm and milled at 300 rpm using alumina balls (99.94%). The milling times were varied to 6, 12, and 18 h. During milling, some of the fibers that were not pulverized became aggregated and formed fiber balls, which are continuously packed and decreaseed pulverization efficiency. To avoid this, the mPCFs were enmeshed through a 230-mesh (under 63 µm) and prepared as sorted fine fibrous powder.

**Figure 1:**
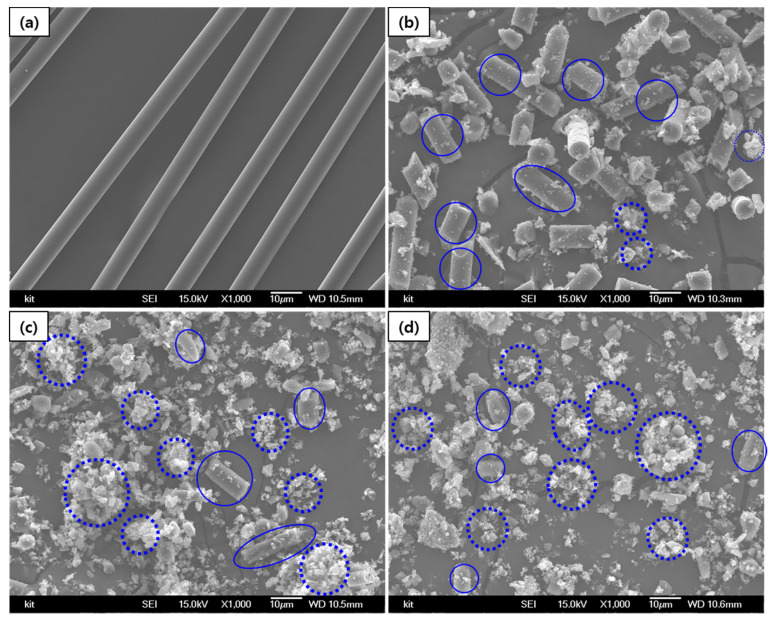
SEM images of mPCFs (×1000). (**a**) Desized carbon fiber and ball milling times of (**b**) 6 h, (**c**) 12 h, and (**d**) 18 h.

**Figure 2:**
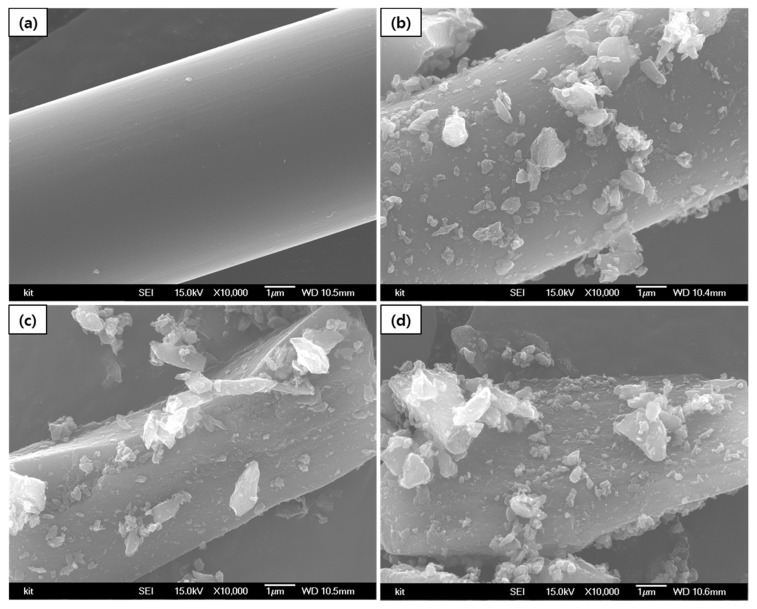
SEM images of mPCFs (×10,000). (**a**) Desized carbon fiber and ball milling times of (**b**) 6 h, (**c**) 12 h, and (**d**) 18 h.

After ball milling for 6, 12 and 18 h, the particle size of the mPCFs (D_50_) decreased to 10.63 µm, 3.25 µm, and 1.83 µm, respectively. Figure 1 and Figure 2 show the SEM images of the mPCFs depending on the ball milling time.

The mPCFs shown in Figure 1 include partially-milled Carbon Fiber (pmCF, solid line), which retains the surface and cross-sectional shape of a conventional fiber, and milled Carbon Fiber (mCF, dotted line) that has been completely pulverized and has lost the fibrous shape. After 6 h of milling time, the mPCFs were mostly pmCF. The ratio of pmCF decreased and that of mCF increased as the milling time was increased.

Figure 1 and Figure 2 show that a portion of the pmCF that was milled for 12 h had a complete fibrous shape (Figure 1c), but most of the pmCF had the shape of broken fibers (Figure 2c). The SEM images of the sample that was milled for 18 h showed no pmCF with a complete fibrous shape, and all the fibers were broken.

The 10,000 enlarged images in Figure 2 shows that the surface of the fiber was broken after 12 h, and mCF particles attached to the pmCF were observed. Since the mPCFs were found to be a mixture of fiber and powder, the measurements were performed by differentiating pmCF from mCF by using the optical images obtained from the Raman analysis.

### 2.3. X-ray Diffraction and Conductivity

The crystallinity dependence on ball milling time was analyzed by XRD (X-MAX/2000-PC, Rigaku). The wavelength of the X-ray target (Cu-K_α1_) used in the XRD analysis was 1.5406 Å, and the XRD spectrum was obtained by 2θ continuous scanning in a scan range of 10 to 60° at a scan rate of 1°/min [13,14]. The measurement was performed three times for each sample.

After removing the background, the (002) peak was separated into two bands (LDCC and MDCC) by Gaussian fitting in order to calculate *d*_002_ and *L_c_*. The 2θ and the full width at half maximum value obtained from each band and peak were used to calculate planar spacing using the Bragg equation, and the Lc and La were calculated with the Scherrer equation [13,14].

The dependence of the electric conductivity of the mPCFs on the milling time was measured using a powder resistivity measurement system (HAN TECH Co. Ltd., Gunpo, Korea). Pulverized mPCF was packed into a mold possessing a diameter of 20 mm, and the resistivity was measured by 4-point probe and converted into electric conductivity. R. Holm et al. [15,16] explained that the electric conductivity of powder is lower than that of individual particles because the interface between particles introduces additional resistance when charges are transported. Applying pressure during the measurement of the electric conductivity of powder increases the electric conductivity, by increasing the contact area between particles. In the present study, the electric conductivity of the mPCF powder was measured by varying pressures between 1.29 and 51.6 MPa in order to find the trend. In this paper, we used the measurement values obtained at 51.6 MPa.

### 2.4. Raman Spectroscopy

The crystallinity dependence on milling time was measured using a Raman spectroscopy (Raman, System 1000, Renishaw, Wotton-under-Edge, UK). Raman spectroscopy was performed with an Ar-ion laser at a wavelength of 541 nm [17].

Researchers have presented various values for the constant *C* in the domain size of *L_a_* = (*C/R*) according to laser type and wavelength. Knight and White [18] employed an Ar-ion laser at a wavelength of 514.4 nm and calculated that the crystallite *L_a_* in a single crystal of graphite was (*44/R*). M. Endo [2] prepared milled mesophase pitch-based carbon fiber and analyzed the crystallinity by Raman spectroscopy with a HeNe laser at a wavelength of 632.8 nm, reporting that the calculated crystallite *L_a_* was (*120/R*). Roh [19] analyzed the structure of activated carbon fiber by Raman spectroscopy with an Ar-ion laser at a wavelength of 514.5 nm and reported that the calculated *L_a_* was (*20/R*).

In the present study, we analyzed the change in crystallinity by measuring the peak intensity ratio, *R (=I_D_/I_G_)*, of the D-band at 1360 cm^−1^ relative to the G-band at 1580 cm^−1^ [11,17]. The intensity ratio of the peaks was calculated without modifying the baseline of the Raman spectra. We separately analyzed the surface crystallinity of the fine mCF powder and that of the pmCF, which was not completely pulverized. The broken fibrous shapes observed in Figure 1; Figure 2c,d were all considered to be pmCFs, and the measurement was performed by ignoring the mCF attached to the pmCF surface. Three Raman spectra were obtained from each pmCF and mCF, and their dispersions are presented.

### 2.5. TEM

The specimens used for TEM (JEOL, JEM, and 2100) analysis were prepared using the common method for preparing fiber specimens. The fibers were arranged in one direction, cured in an epoxy resin, and kept in a vacuum oven at 70 ℃ for 24 h. After that, the resulting specimens were cut by using a diamond knife attached to Cryo-Ultramicrotome (RMC, PTPC&CRX). The cut fiber specimens were mounted on a Cu-grid and observed at an acceleration voltage of 200 kV. The grid images were obtained at a magnification of 800,000 by under-focusing after determining the conditions under which the crystallite layers could be clearly viewed. We tried to observe the surface and the inside of the fiber specimens separately, but it was impossible because the sampling with the diamond knife was carried out randomly. The lattice images and the diffraction patterns (DP) were obtained at a magnification of 800,000. We observed at least 3 points of a sample that were considered to be homogeneous [20,21]. Only the brightness and contrast of the images were adjusted without any special image processing.

## 3. Results and Discussion

### 3.1. X-ray Diffraction and Conductivity

**Figure 3:**
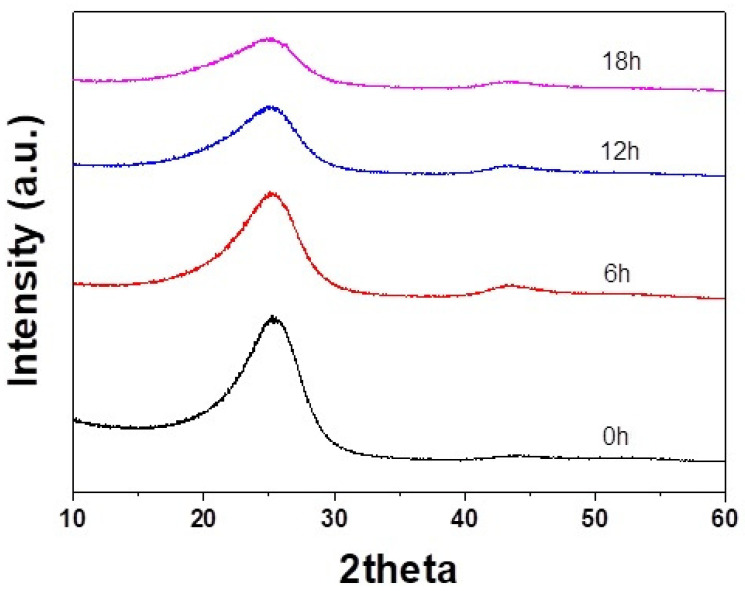
XRD patterns of mPCFs depending on milling time.

Table 1 and Figure 3 show the change of the XRD structural factors of the mPCFs depending on the milling time. The values of the structural factors are the mean values of three measurements from each specimen. In the desized carbon fiber, the *d*_002_ and *L_c_* values at the LDCC band were 3.750 Å and 12.77 Å, respectively, and those at the MDCC band were 3.466 Å and 24.33 Å, respectively. As the milling time increased, the *d*_002_ increased, and the *L_c_* decreased for both the LDCC and MDCC bands. After 18 h of milling time, the *d*_002_ and *L_c_* values of the LDCC band were 3.804 Å and 11.33 Å, respectively, and those of the MDCC band were 3.503 Å and 23.47 Å, respectively. The La of the desized carbon fiber was 51.63 Å. With increasing milling time, it decreased to 50.27 Å at 12 h and then increased to 52.70 Å at 18 h [13].

Table 2 shows the electric conductivity of the mPCFs measured by using the powder resistivity measurement system under a load of 51.6 MPa. The electric conductivity of the desized carbon fiber was 98.1 S/cm. As the milling time increased, the electric conductivity decreased to 6.54 S/cm at 18 h. The decrease in electric conductivity was greatest between 0 and 6 h of milling time. The ball milling process decreased the fiber length, and the reduction in internal crystallinity acted as defects, reducing the electric conductivity.

### 3.2. Raman Spectroscopy

**Figure 4:**
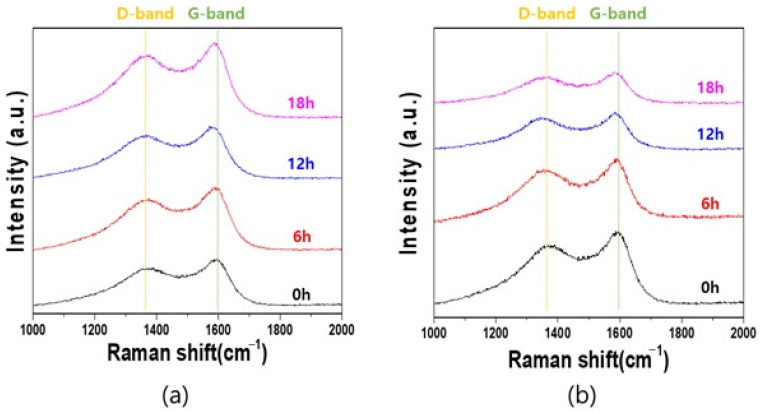
Raman spectra of mPCFs, (**a**) pmCF, and (**b**) mCF.

Figure 4 shows the Raman spectra of the pmCF and mCF depending on the milling time. As the milling time increased, the peak intensity of the G-band of the mCF decreased, while that of the D-band increased.

**Figure 5:**
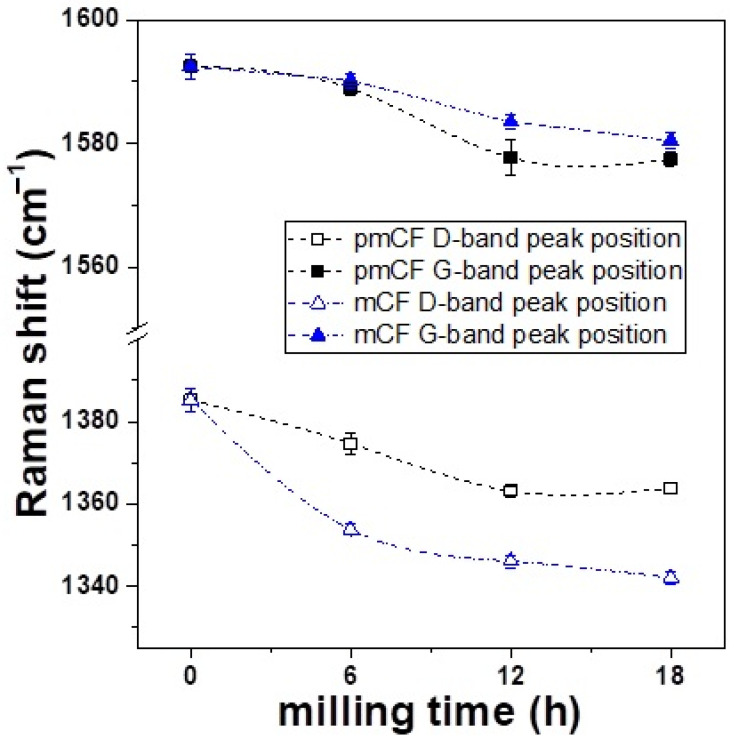
Peak position change of the first-order D and G bands.

Figure 5 shows that the ball milling process affected the position of the Raman bands. In the G-band, the Raman peak positions of the pmCF and mCF were almost similarly shifted to a lower value. However, a closer look at the shift revealed a slight difference. At the milling time of 6 h, the Raman band position of the pmCF, which still retained the complete shape, also shifted. At the milling time of 12 h, the position shift of the milled pmCF was greater than that of mCF. This suggests that slight disordering was also occurring on the surface of the pmCF that was not pulverized to powder and, thus, maintained a complete shape. The disordering may have been larger in the mCF that was further pulverized and broken.

In addition, the Raman peak position in the D-band of the mCF shifted to a lower value at an earlier milling time than in the D-band of the pmCF. This is possibly due to the poor organization of carbon materials (i.e., more disorder) [22]. The change in the gradient of the D-band (peak position shift) of the mCF at 6 h of milling time was very large, but the change in gradient became similar for milling times longer than 6 h. This suggests that the change in the gradient of the D-band was large within 6 h of milling time because the internal crystals were severely damaged in the early milling process. The continued ball milling process may have turned the pmCF into mCF, and a similar magnitude of stress may have been applied to the ordered phase and the disordered phase. This finding may be associated with the abovementioned XRD results, which showed a drastic change in the *d*_002_ and *L_c_* in the LDCC band of the sample at 6 h of ball milling time. These results verify that the increase in *d*_002_ and the decrease in *L_c_* in the early milling process are related to the damage of the internal crystals [8].

**Figure 6:**
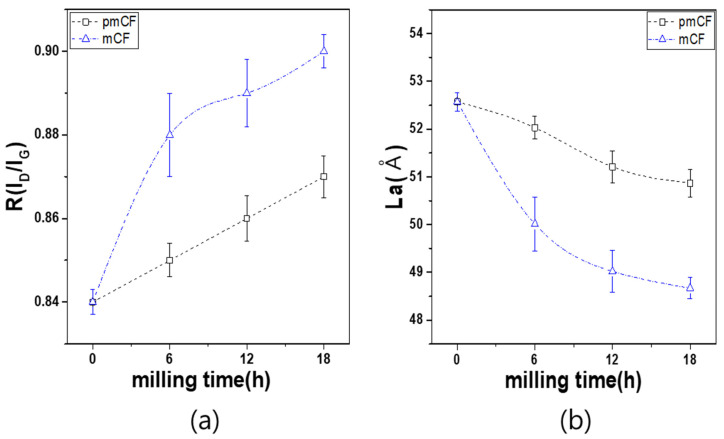
Raman spectroscopic results of mPCFs depending on milling time. (**a**) *R* and (**b**) *L_a_*.

Table 3 and Figure 6 show the *R* and *L_a_* of the pmCF and mCF depending on the milling time. Figure 6a shows that the *R* value of the desized carbon fiber was 0.84, and the value of both the pmCF and mCF increased with the increase in milling time. At 18 h of milling time, the *R* value of the pmCF was 0.87 and that of the mCF was 0.90. While the *R* value of the pmCF linearly increased with the increase in milling time, that of the mCF rapidly increased within 6 h and then further increased slowly.

The Raman crystallite size is expressed as *L_a_ = (C/R)*, and the most appropriate value of the constant *C* was 44 in comparison with the *L_a_* value from the XRD data. Therefore, we used *L_a_ = (44/R)* for the calculation in the present study. Figure 6b shows that the Raman crystallite size *L_a_* decreased from the initial value of 52.57 Å to 50.87 Å in pmCF, and to 48.67 Å in mCF at 18 h of milling time. The *L_a_* of pmCF slowly decreased, while that of the mCF rapidly decreased within 6 h and then slowly decreased similar to the pmCF.

**Figure 7:**
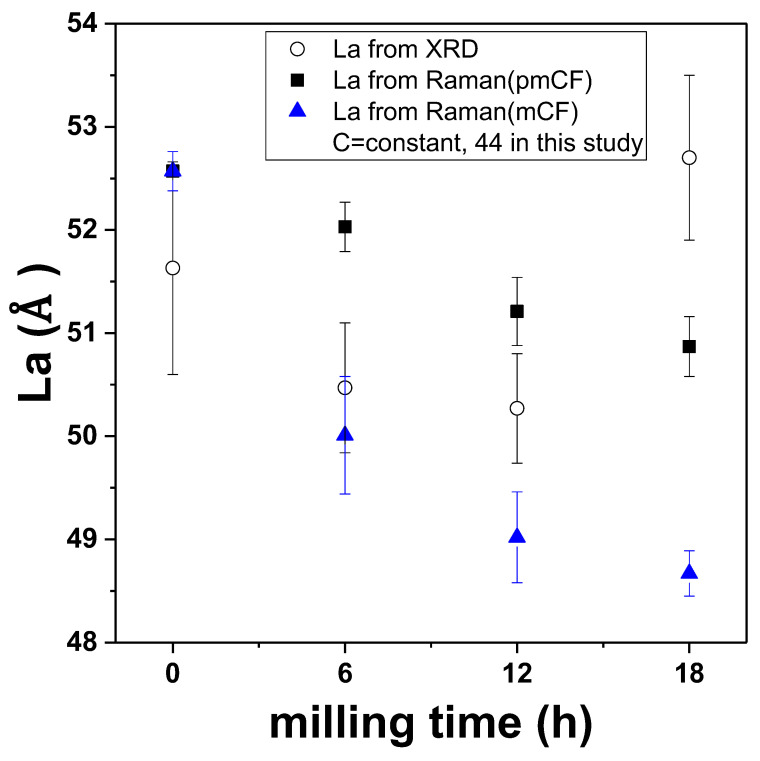
Correlation of *L_a_* values from Raman and XRD results.

Figure 7 compares the *L_a_* values obtained from XRD with those obtained from Raman spectroscopy. The Raman *L_a_* value was similar to the XRD *L_a_* value during the early stage of the milling process. At 12 h of milling time, the *L_a_* value was different between the Raman data and the XRD data. The difference may be due to the broken pieces of the pmCF that still remained even after 12 h of milling time, as can be observed from the SEM images.

Since Raman spectroscopy is limited to the surface of the samples, the rate of the *L_a_* decrease was slower in the larger pmCF samples than in the mCF samples. However, the crystallinity gradually decreased in both samples as the milling process continued.

By contrast, the XRD data were obtained from not only the surface of the mCF and pmCF samples but also their entire particles, including the inside. Therefore, the XRD measurement data may have been affected by the presence of the considerable volume of pmCF particles that remained, even after 18 h of milling time.

Assuming that the increase in the *L_a_* at the milling times of 12 h and 18 h is because of a change inside the pmCF particles (XRD results), the growth of the *L_a_* in the pmCF particles may be due to the ball milling process. In other words, the crystal growth may have been caused by mechanical stress. Obviously, milling gradually reduces the crystallinity of fiber surface and pulverizes fibers. Our finding about the change inside the particles by mechanical stress in a milling process has never been reported in the field of carbon materials.

A similar example was found in mechanical alloying, that occurs in the pulverization of a metallic material. P. S. Gilman et al. [23,24] studied the mechanical alloying of metal powder and reported that the ball-powder-ball collision affects the particles. They explained that as the particles are flattened and overlapped during ball milling, atomically clean interfaces come into close contact with one another to form composite particles, as in cold welding. They referred to the formation of the composite particles as mechanical alloying, which is a representative example of the homogenizing and refining of particles by mechanical stress. M. Mhadhbi et al. [21] also reported that crystallite size was increased by the mechanical alloying of Fe and Al.

Such mechanical alloying may be applied to carbon fibers impacted by balls in a milling process. The crystalline structure of carbon materials is composed of layers bonded by the weak van der Walls force, and their bonds may be easily broken by the impact of the balls. However, the lattices have strong sp^2^ covalent bonds, which are relatively resistant to the balls’ impact. Although the fiber surface is easily broken and pulverized by the balls, the unbroken internal part of the fiber may undergo mechanical alloying by the mechanical stress (mPCFs at 18 h of milling time) and grow into fine crystal grains.

Therefore, in the present study, the internal part of the mPCFs fiber may have undergone ‘mechanical alloying’ by the long-term ball-fiber collisions (mechanical stress) during the ball milling process, and the La may have been increased after 18 h of milling as a result (XRD results).

### 3.3. TEM

**Figure 8:**
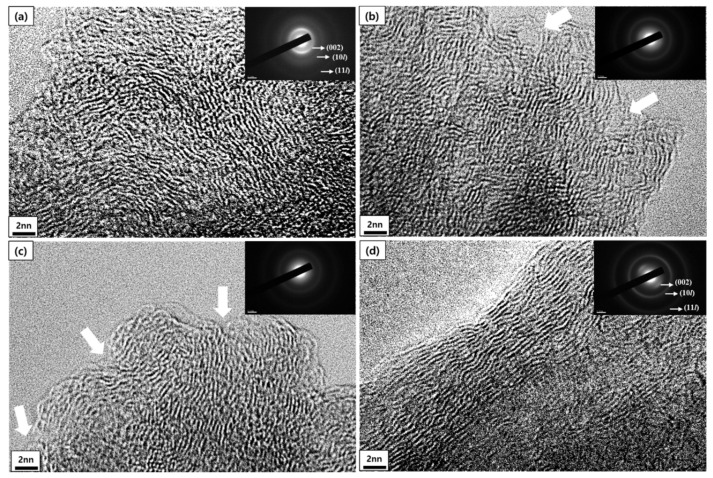
TEM lattice image and diffraction pattern (×800 k) of mPCFs depending on milling time: (**a**) desized carbon fiber; ball milling times of (**b**) 6 h mPCFs, (**c**) 12 h mPCFs, and (**d**) 18 h mPCFs.

Figure 8 shows the diffraction patterns and the TEM lattice images of the mPCFs depending on the milling time. The images were obtained from at least three randomly selected points that were considered most general. As shown in Figure 8a, the (002) diffraction pattern was difficult to observe accurately due to the strong light of the transmitted beam; thus, the TEM lattice images were very difficult to obtain. Since carbon fibers laminated in parallel in the A–B–A–B form has a turbostratic structure rather than a three-dimensional graphite structure, the (10*l*) and (11*l*) diffraction patterns are not clearly distinguished.

Figure 8b shows that the (002) diffraction pattern of the mPCFs at 6 h of milling time was not as distinctive as that of the desized carbon fiber, indicating that crystallinity had decreased in comparison with the starting fiber materials. Figure 8b shows some crystallites that were bent by the ball impact and some adjacent crystallite layers that were disconnected (white arrows).

Figure 8c shows that the (002) diffraction pattern of the fibers became vaguer at 12 h of ball milling. The vaguer (11*l*) diffraction pattern indicates that the lamination of the crystallites became worse. The disconnection between adjacent crystallite layers, already found at 6 h of milling time, was more outstanding at 18 h of milling time.

Figure 8d shows the diffraction pattern of the fiber that was milled for 18 h, and it was more distinctive than that of other fiber samples. The more distinctive diffraction pattern suggests that higher crystallinity resulted from the better organization of the external crystallite layers.

The lattice images and diffraction patterns show that the crystallites became disordered at 12 h of ball milling time, but the crystals were again developed at 18 h of milling time. This is consistent with the XRD results that the *L_a_* value decreased at 12 h of milling time and then increased at 18 h.

## 4. Conclusions

We investigated the effect of ball milling time on fiber structure in the preparation of mPCFs and obtained the following conclusions.

1. Structural Analysis by XRD

The crystallite size, *L_a_*, obtained from the XRD spectra decreased at 12 h of ball milling time and then drastically increased at 18 h. This suggests that the *L_a_* decreased during the early stage of the milling process as the fibrous surface was broken and then, as the milling time was further increased, *L_a_* was increased by internal crystal growth induced by the impact energy from the balls, as in the case of mechanical alloying.

2. Structural Analysis by Raman Spectroscopy

The Raman data showed that the disorder in the pmCF and mCF samples gradually increased, and *L_a_* decreased as the milling time was increased. Since Raman spectroscopy analyzes the crystallinity on the fibrous surface, the observed decrease in the *L_a_* value may be due to the continuous breaking of the fibrous surface by the ball milling. The crystallinity decreased more in the mCF samples, which were pulverized more. In addition, the calculation of the *L_a_* showed that the appropriate value of the constant *C* was approximately 44 for the mPCFs.

3. Structural Analysis by TEM

The lattice images and diffraction patterns showed that the crystallites became disordered at 12 h of milling time, and then crystals developed again at 18 h of milling time. This finding is consistent with the trend in *L_a_* found in the XRD results. Although accurately distinguishing the pmCF from the mCF may not be possible due to limitations, separate sampling of the pmCF and mCF may be a good method for analyzing the structures.

Based on this study, it is suggested that mCF can be used as an anode material for batteries and as a filler material for artificial graphite blocks in the future. In particular, it is expected to be useful for inducing the development of crystallinity on the surface of mCF with a longer milling time from the TEM results.

## Figures and Tables

**Table 1:** XRD structural factors of mPCFs depending on milling time [13].

Milling Time (h)	002 Peak (LDCC)			002 Peak (MDCC)			10*l* Peak	
	2θ	*d*_002_ (Å)	*L_c_* (Å)	2θ	*d*_002_ (Å)	*L_c_* (Å)	2θ	*L_a_* (Å)
0	24.38	3.750	12.77	25.68	3.466	24.33	44.23	51.63
6	23.43	3.793	11.57	25.45	3.497	23.67	43.68	50.47
12	23.42	3.795	11.37	25.46	3.496	23.30	43.67	50.27
18	23.37	3.804	11.33	25.40	3.503	23.47	43.50	52.70

**Table 2:** Electric conductivity of mPCFs depending on milling time.

Milling Time (h)	Press (MPa)	VR (Ω∙cm)	Conductivity (S/cm)
0	51.6 (2000 kg)	0.0102	98.1
6		0.0502	19.9
12		0.0924	10.8
18		0.1530	6.54

**Table 3:** Analysis of peaks of mPCFs depending on milling time.

	pmCF						mCF					
Time (h)	Peak Position		Peak Intensity		*R* (=*I_D_/I_G_*)	*L_a_* (Å)	Peak Position		Peak Intensity		*R* (=*I_D_/I_G_*)	*L_a_* (Å)
	D-Band	G-Band	D-Band	G-Band			D-Band	G-Band	D-Band	G-Band		
0	1385.3	1592.5	2265.4	2706.6	0.84	52.57	-					
6	1374.7	1588.9	3145.4	3719.2	0.85	52.03	1353.8	1590.2	2414.8	2744.8	0.88	50.01
12	1363.2	1577.7	2665.5	3102.3	0.86	51.21	1346.1	1583.5	1328.4	1480.1	0.89	49.02
18	1363.9	1577.4	3870.1	4474.6	0.87	50.87	1342.1	1580.5	1185.0	1310.7	0.90	48.67

## Data Availability

Data available on request from the corresponding authors due to restrictions eg privacy or ethical.
